# A Magnetic Adsorbent for the Removal of Cationic Dyes from Wastewater

**DOI:** 10.3390/nano8090710

**Published:** 2018-09-10

**Authors:** Yumei Ji, Chenguang Ma, Jie Li, Haiyan Zhao, Qianqian Chen, Mingxue Li, Hongling Liu

**Affiliations:** Henan Key Laboratory of Polyoxometalates, Institute of Molecular and Crystal Engineering, College of Chemistry and Chemical Engineering, Henan University, Kaifeng 475004, China; 15090606771@163.com (Y.J.); 104753160773@vip.henu.edu.cn (C.M.); hedalj@163.com (J.L.); 15890943739@163.com (H.Z.); 18739970393@163.com (Q.C.)

**Keywords:** nanocomposite particles, polyoxometalates, adsorption, magnetic properties

## Abstract

In this article, a study was presented on the adsorption activity of a new nanocomposite particle **Fe_3_O_4_@1**, which was synthesized by combining [Cu(HL)_2_]_2_H_2_[P_2_Mo_5_O_23_]·10H_2_O (**1**) (HL = 2-acetylpyridine semicarbazone) and Fe_3_O_4_ nanoparticles. Transmission electron microscopy and X-ray powder diffraction analyses revealed that **Fe_3_O_4_@1** possessed high crystallinity with an average particle size of 19.1 nm. The adsorption activity of the as-prepared **Fe_3_O_4_@1** was investigated by photometrically monitoring the removal of methylene blue, rhodamine B, safranine T, gentian violet, fuchsin basic, and methyl orange from aqueous solutions. Significantly, we could easily separate **Fe_3_O_4_@1** from the reaction media by applying an external magnet. Furthermore, the recycling performance was observed using methylene blue, revealing the recyclability and high stability of **Fe_3_O_4_@1**. It was shown that **Fe_3_O_4_@1** is a promising candidate material for adsorbing cationic dyes in aqueous media.

## 1. Introduction

With the development of human society, the discharge of a great deal of wastewater has come to pose a significant threat to the hydrographic environment and public health because of the toxicity and carcinogenicity of substances present in this material [1,2,3]. Dyes have been widely used in various industries, such as paper production, textile production, leather tanning, food technology, and hair coloring. It is estimated that more than 100,000 commercially available dyes are produced at a rate of over 7 × 10^8^ kg every year [4]. The discharge of dyes into the environment causes both toxicological and esthetic problems [5]. There are diverse toxic substances and organic compounds in this wastewater, such as methylene blue (MB), rhodamine B (RhB), safranine T (T), gentian violet (GV), fuchsin basic (FB), and methyl orange (MO), which are harmful to fish and other aquatic organisms [4,5,6]. Until now, more than 15% of dye loss is due to incomplete depletion of dye washing operations and the dyeing process [7]. The discharge of these dyes without any treatment threatens aquatic ecosystems and human health. It is highly desirable to seek proper treatment strategies to eliminate dye residues from wastewater systems. Hence, appropriate treatment strategies are necessary to eliminate dyes from wastewater.

At present, two main strategies are extensively explored. One is photocatalytic degradation [8], which is an advanced oxidation process that mainly occurs under light irradiation and with suitable photocatalytic materials. However, the photocatalytic activity of photocatalytic materials for the degradation of dyes present in water depends largely on the band gap, surface area, and generation of electron-hole pairs [9]. The other strategy is the adsorption process. It has been demonstrated that adsorption is a suitable and effective approach because of its simple design, facile working conditions, low energy requirements, and insensitivity to toxic substances [10]. The adsorption materials have attracted considerable interest by using nanomaterials as efficient adsorbents in aqueous media [11,12].

In the last few years, polyoxometalates/nanoparticles (POMs/NPs) have been successfully used for dye degradation [13]. Due to its unique properties, the combination of polyoxometalates with nanoparticles has attracted wide attention. On the one hand, POMs have aroused considerable interest in catalysis, redox reactions, medicine, magnetism, materials chemistry, electrochemistry, and photochemistry, due to their oxo-enriched surfaces, high electronegativity, controllable shape, tunable acid-base properties, and many active coordination sites [14]. On the other hand, magnetic Fe_3_O_4_ nanoparticles are most widely known as environmentally-friendly materials for industrial-scale synthesis of fine chemicals, due to their unique physical and chemical properties [15]. The combination of POMs and Fe_3_O_4_ nanoparticles at the molecular scale will be conducive to the resulting complex with new features and multiple special functionalities, which are different from those of the individual ingredients alone. Therefore, the purpose of this work was to use polyoxometalates and nanoparticles to assess the adsorption potential of nanocomposites for organic dyes. Appropriate precursors and synthetic methods are utilized to synthesize the target products.

Herein, we present an example of **Fe_3_O_4_@1**, which has selective adsorption behavior for cationic organic dyes: MB, RhB, T, GV, and FB. The **Fe_3_O_4_@1** could be easily isolated from sample solution by applying an external magnetic field. **Fe_3_O_4_@1** exhibited stability and recyclability. These results proved that the **Fe_3_O_4_@1** could be of interest as a magnetic adsorbent.

## 2. Materials and Methods

### 2.1. Materials

All reagents and solvents were purchased commercially and used without further purification. Copper(II) perchlorate hexahydrate (Cu(ClO_4_)_2_·6H_2_O, 98%), sodium molybdate dihydrate (Na_2_MoO_4_·2H_2_O, 99%) and phosphoric acid (H_3_PO_4_, 85%) were purchased from J&K Scientific Ltd. (Beijing, China) Poly(ethylene glycol)-block-poly(propylene glycol)-block-poly(ethylene glycol) (PEO-PPO-PEO, Mr = 800), iron(III), acetylacetonate (Fe(acac)_3_, 99.9%), 1,2-hexadecanediol (C_14_H_29_CH(OH)CH_2_(OH), 90%), octyl ether (C_8_H_17_OC_8_H_17_, 99%), DMF (HCON(CH_3_)_2_, 99.5%) and solvents such as hexane and ethanol were purchased from Aldrich (Shanghai, China). 

### 2.2. General Procedures

The structures of nanoparticles were analyzed by transmission electron microscopy (TEM, JEOL 2010F, JEOL Ltd., Tokyo, Japan) including the mode of high resolution (HRTEM) and X-ray powder diffraction (XRD, X’Pert Pro, Bruker, Karlsruhe, Germany). Magnetic properties were determined by a vibrating sample magnetometer (VSM, Lakeshore 7300, Quantum Design, San Diego, CA, USA). Elemental analyses (C, H and N) were implemented on a Flash 2000 analyzer (Elementar, Hessia, Germany). Inductively coupled plasma (ICP) analysis was performed on a optima 2100DV (PerkinElemer, Waltham, MA, USA). The infrared (IR) spectrum was obtained on a VERTEX 70 (Bruker, Karlsruhe, Germany) using KBr pellets in the range of 4000–500 cm^−1^. The UV–Vis absorption spectrum was recorded with a TU–1900 spectrometer (Beijing Purkinje General Instrument Co., Ltd., Beijing, China) at room temperature. X-ray photoelectron spectroscopy (XPS) was carried out on a Thermo ESCALAB 250XI photoelectron spectrometer (ThermoFisher Scientific, Waltham, MA, USA) with Al Kα X-ray as the excitation source.

### 2.3. Synthesis

Synthesis of compound [Cu(HL)_2_]_2_H_2_[P_2_Mo_5_O_23_]·10H_2_O (**1**):

A 25 mL solution (V_methanol_/V_water_ = 2/3) containing Cu(ClO_4_)_2_·6H_2_O (0.093 g, 0.25 mmol) and 2-acetylpyridine semicarbazone (0.098 g, 0.5 mmol) was stirred at 60 °C for 30 min. After the solution was cooled to room temperature, it was added to a 10 mL aqueous solution of Na_2_MoO_4_·2H_2_O (0.242 g, 1.0 mmol) with one drop of H_2_O_2_, and the pH was maintained at approximately 3.0 by adding concentrated H_3_PO_4_ under continuous stirring. The mixture was stirred for another 30 min and then cooled and filtered. The filtrate was placed at room temperature for slow evaporation. Blue crystals of **1** were isolated after 3 days. Yield: approx. 59% (based on Cu). Elemental analysis for C_32_H_58_Cu_2_Mo_5_N_16_O_37_P_2_: calcd. C 19.90, H 3.02, N 11.60, Mo 24.87, Cu 6.61; Found: C 19.94, H 3.04, N 11.63, Mo 24.89, Cu 6.59. IR (KBr, cm^−1^): 3394 (m), 3186 (w), 1667 (s), 1602 (w), 1528 (m), 1471 (m), 1440 (w), 1378 (s), 1332 (w), 1304 (w), 1269 (w), 1201 (m), 1163 (w), 1115 (m), 1055 (m), 1008 (m), 922 (s), 903 (s), 782 (w), 697 (s), 569 (m), 552 (m), 503 (w).

Synthesis of **Fe_3_O_4_@1**:

**Fe_3_O_4_@1** was obtained through an ultrasonic method in a 25 mL beaker. Fe_3_O_4_ (7.5 mg), which were synthesized according to the method reported in the literature [16,17], and **1** powder (50 mg) were added to a beaker that contained water (5 mL) and ethanol (5 mL), and then a uniform suspension was obtained via ultrasound for approximately 10 h. The resulting magnetic products, **Fe_3_O_4_@1**, were collected from the suspension by using a magnet, and washed with water several times.

### 2.4. Crystallography

A high-quality single crystal was carefully selected under an optical microscope. Crystallographic data were collected with a Bruker SMART-CCD APEX II diffractometer (Bruker-AXS, Karlsruhe, Germany) with a graphite-monochromator with Mo *K*α radiation (*λ* = 0.71073 Å). The structures were solved via direct methods, and refined by full-matrix least squares on *F*^2^ with anisotropic displacement parameters for all nonhydrogen atoms using SHELXTL [18]. Hydrogen atoms were added in idealized geometrical positions. The crystal data, experimental details, and refinement results were listed in Table 1. The CCDC number for **1** is 128509.

### 2.5. The Experimental and Procedures Adopted for the Adsorption

The adsorption activities of the nanocomposites were performed in the dark by measuring the adsorption rate of different dyes solutions at room temperature. The typical process is as follows: 2 mg of **Fe_3_O_4_@1** was suspended in 10 mL of a 15 mg L^−1^ MB aqueous solution. The solutions were magnetically stirred in the dark. At several time intervals, 4 mL of sample was removed, centrifuged several times to separate **Fe_3_O_4_@1**, and a clear solution was obtained for the UV–Vis analysis.

## 3. Results and Discussion

### 3.1. Crystal Structure Description of Compound ***1***

**Figure 1:**
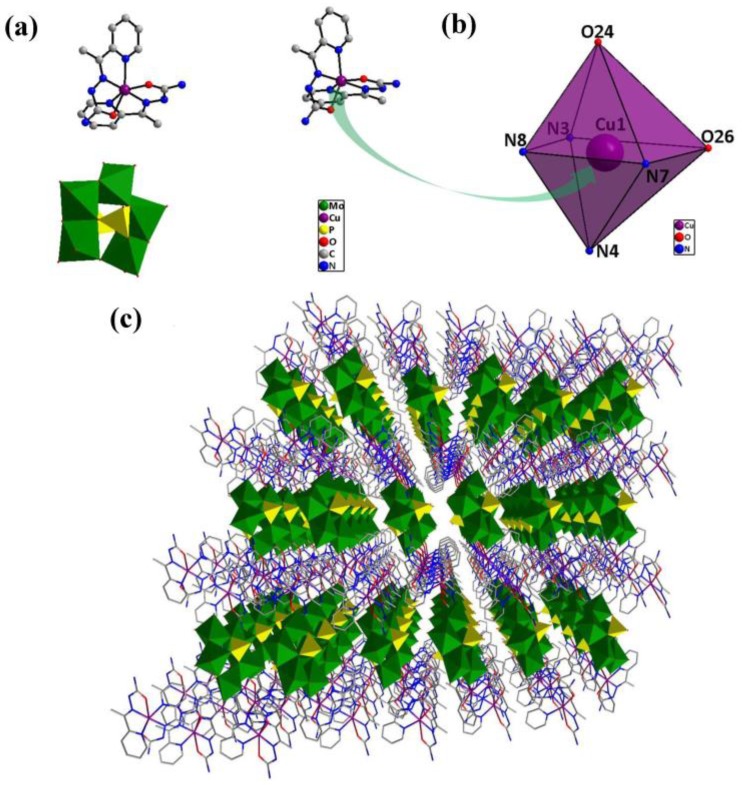
(**a**) Structure of compound **1**; (**b**) structure of the six-coordinate distorted octahedral geometry around copper and (**c**) polyhedral/wire-stick representation of the 3D network along the *a*-axis.

The single-crystal X-ray diffraction analysis reveals that compound **1** is triclinic. Compound **1** (inset of Figure 1a) consists of one [P_2_Mo_5_O_23_]^6−^ unit [19], two [Cu(HL)_2_]^2+^ coordination groups and ten lattice water molecules. Each of two crystallographical independent copper(II) ions with a similar coordination environment adopts a six-coordinate distorted octahedral geometry (Figure 1b). The crystallographic analysis showed that each Cu(II) is coordinated by two O atoms and four N atoms from two HL ligands, with Cu–N bond lengths of 1.949(3)–2.200(4) Å and Cu–O bond lengths of 2.096(3)–2.298(3) Å. Figure 1c exhibits the polyhedral/wire-stick representation of the 3D network along the *a*-axis.

### 3.2. XRD Patterns

**Figure 2:**
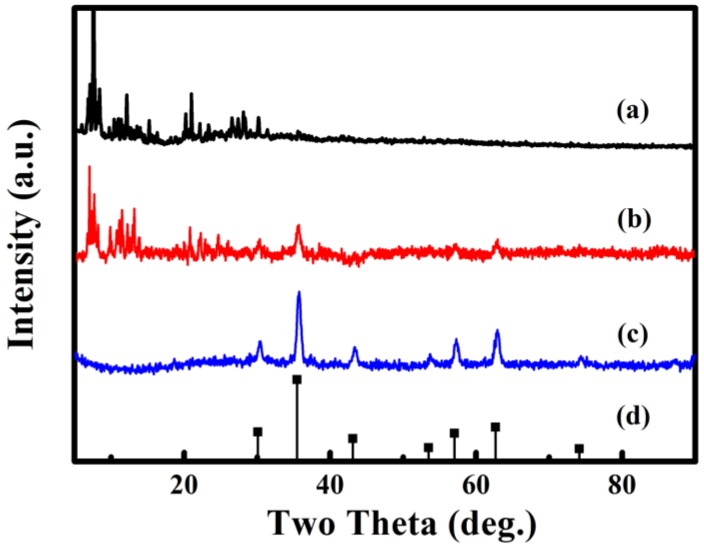
XRD diffraction patterns of (**a**) **1**, (**b**) **Fe_3_O_4_@1**, (**c**) Fe_3_O_4_ and (**d**) Fe_3_O_4_ standard card.

According to Figure 2, the structures of **1**, **Fe_3_O_4_@1** and Fe_3_O_4_ are analyzed by XRD. Figure 2a shows the diffraction pattern of **1**. Figure 2c shows the diffraction pattern obtained from Fe_3_O_4_ matched to the standard diffraction peaks (Figure 2d) of the corresponding Fe_3_O_4_ (JCPDS No. 88-0315), the diffraction peaks located at 30.15°, 35.52°, 43.17°, 53.56°, 57.09°, 62.70° and 74.18° are indexed to the (220), (311), (400), (422), (511), (440) and (533) planes of the Fe_3_O_4_. Figure 2b exhibits the diffraction pattern of **Fe_3_O_4_@1**, which reveal that **1** and Fe_3_O_4_ are included in **Fe_3_O_4_@1**.

### 3.3. IR Spectroscopy

**Figure 3:**
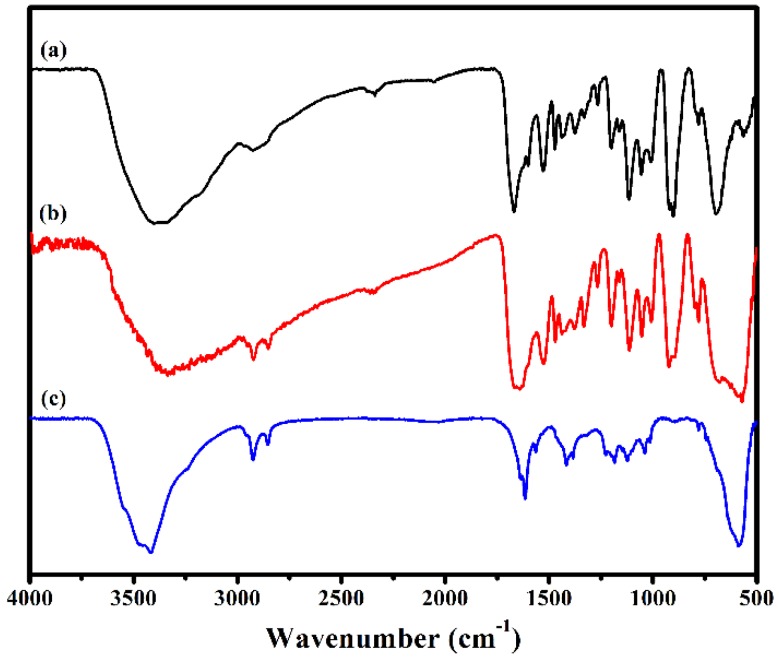
FTIR spectra of (**a**) **1**, (**b**) **Fe_3_O_4_@1** and (**c**) Fe_3_O_4_.

The IR spectra of **1**, **Fe_3_O_4_@1** and Fe_3_O_4_ in the region of 4000–500 cm^−1^ are shown in Figure 3. In the spectrum of **1**, there are two strong characteristic bands at 703 and 928 cm^−1^ assign to the *ν*(Mo–O_b_) and *ν*(Mo–O_t_) modes of [P_2_Mo_5_O_23_]^6−^ [20]. The peak at 1061 and 3421 cm^−^^1^ assigned to P–O and O–H vibration, respectively [21]. The peaks at, 782, 1620 and 3190 cm^−1^ are attributed to the *ν*(C–O), *ν*(C=N) and *ν*(N–H) vibration of HL [22,23]. Fe_3_O_4_ shows a broad peak at 589 cm^−1^ associated with the stretching vibration of Fe–O [17]. These characteristic vibration and bending modes reappear in spectrum of **Fe_3_O_4_@1**. These results are in good agreement with those of the XRD analysis, which further illustrates that Fe_3_O_4_ and **1** exist in **Fe_3_O_4_@1**.

### 3.4. UV-Vis Spectroscopy

**Figure 4:**
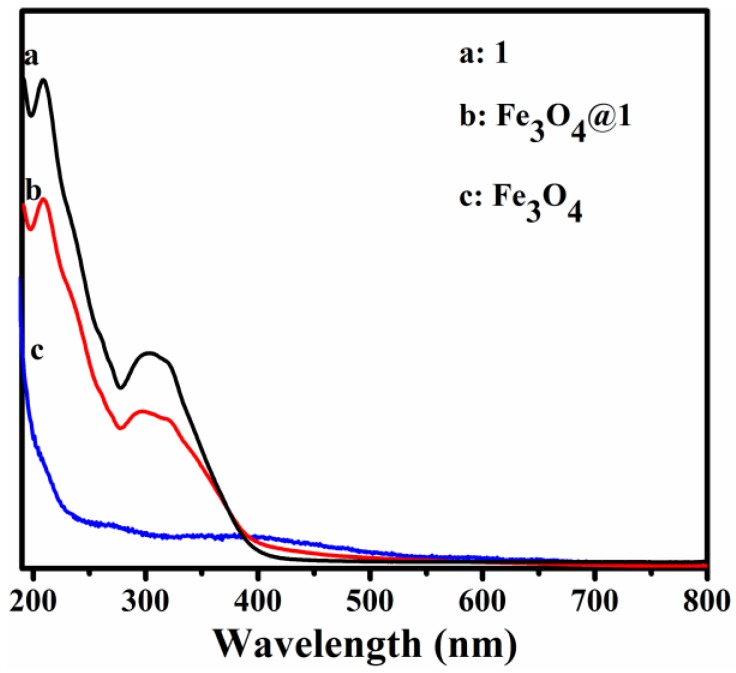
UV-Vis absorbance spectra of (**a**) **1**, (**b**) **Fe_3_O_4_@1** and (**c**) Fe_3_O_4_ dispersed in H_2_O.

The UV-Vis spectra of **1**, **Fe_3_O_4_@1** and Fe_3_O_4_ dispersed in distilled water are shown in Figure 4. Compound **1** shows two peaks at 209 and 316 nm due to the O_t_→Mo and O_b_→Mo charge-transfer bands, respectively (Figure 4a) [20]. Figure 4c shows that the UV spectrum of Fe_3_O_4_ has no obvious absorption bands. Figure 4b shows the peak pattern of **Fe_3_O_4_@1**, which is similar to that of **1**.

### 3.5. XPS Characterization

**Figure 5:**
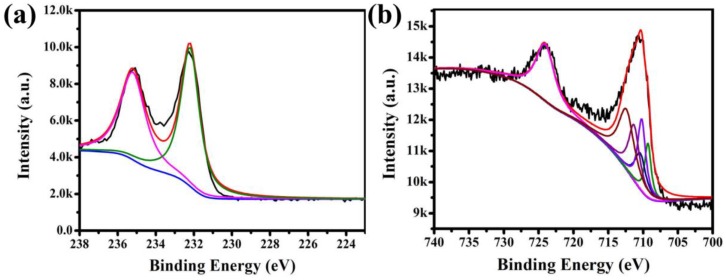
XPS spectra of the **Fe_3_O_4_@1**: (**a**) Mo 3d spectrum and (**b**) Fe 2p spectrum.

The X-ray photoelectron spectra (XPS) for **Fe_3_O_4_@1** were measured in order to identify the elemental composition. The binding energies were calibrated using C 1s peak (284.6 eV). The fingerprint scanning of Mo and Fe in **Fe_3_O_4_@1** was mainly analyzed. The peaks of Mo 3d_3/2_ at 235.2 and Mo 3d_5/2_ at 232.2 eV suggest the existence of Mo and assignation of all the Mo atoms in the +VI oxidation state (Figure 5a) [24]. Figure 5b shows the XPS spectrum of Fe 2p. There is an asymmetrical Fe 2p_3/2_ XPS signal for the samples, which could be divided into three components attributed to the Fe^3+^ species at 710.2, 710.3, 712.4 eV, two components assigned to Fe^2+^ species at 709.3 and 710.4 eV [25,26]. These results further confirmed the existence of Fe_3_O_4_ and **1** in **Fe_3_O_4_@1**.

### 3.6. TEM Morphology and Particle Size Distribution of Fe_3_O_4_ and **Fe_3_O_4_@1**

**Figure 6:**
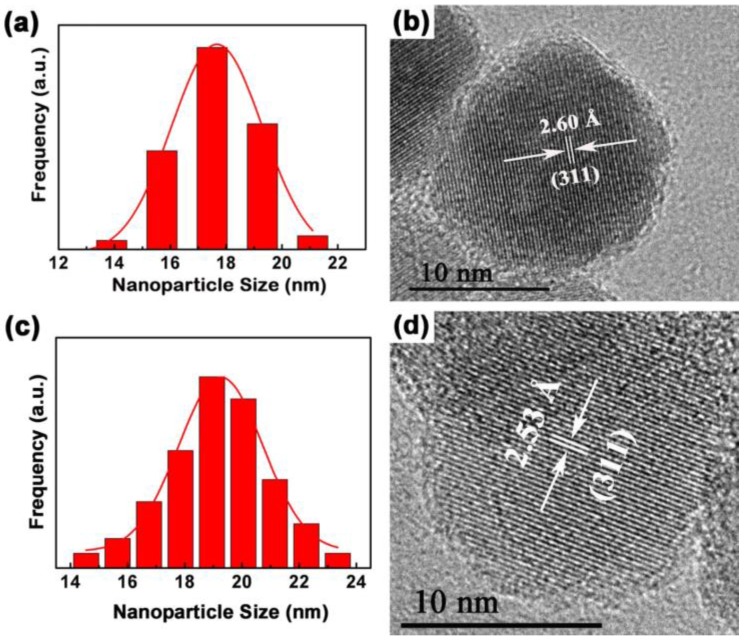
TEM analyses of (**a**) Fe_3_O_4_ and (**c**) **Fe_3_O_4_@1** fitted by a Gaussian function; (**b**) HRTEM of Fe_3_O_4_ and (**d**) **Fe_3_O_4_@1**.

The morphology, nanostructure, particle size and size distribution of the prepared Fe_3_O_4_ and **Fe_3_O_4_@1** were recorded by TEM and HRTEM. As shown in Figure S1, the procured Fe_3_O_4_ and **Fe_3_O_4_@1** are actually uniform and almost spherical in shape. The histograms in Figure 6a,c shows the size distributions of Fe_3_O_4_ and **Fe_3_O_4_@1**, with average diameters of approximately 17.6 nm and 19.1 nm, respectively, which are rationally represented by a Gaussian function. Figure 6b,d represents the HRTEM images of the single Fe_3_O_4_ and **Fe_3_O_4_@1**. The distances of 2.60 Å and 2.53 Å correspond to the (311) and (311) reflections of the Fe_3_O_4_ phase. This further confirms the formation of a stable POM surface corona in **Fe_3_O_4_@1**.

### 3.7. Magnetic Properties of **Fe_3_O_4_@1**

**Figure 7:**
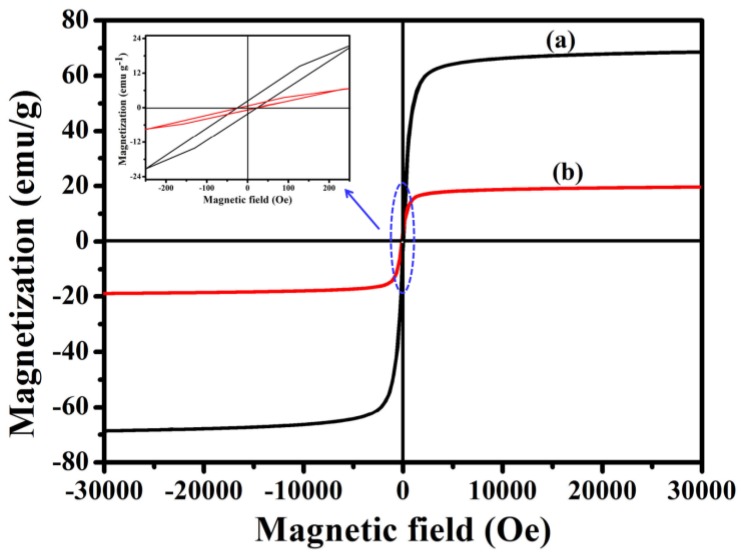
Magnetic measurements of (**a**) Fe_3_O_4_ and (**b**) **Fe_3_O_4_@1**.

In order to investigate the magnetic performance of Fe_3_O_4_ and **Fe_3_O_4_@1**, VSM technology was used. Figure 7 shows the hysteresis curves of Fe_3_O_4_ and **Fe_3_O_4_@1** at 300 K. Notably, **Fe_3_O_4_@1** shows a coercivity of ~0.41 Oe and mass saturation magnetization of ~19.30 emu g^−1^ compared with the values of ~0.22 Oe and ~68.65 emu g^−1^ for Fe_3_O_4_. The decrease in mass saturation magnetization may result from the contribution of the non-magnetic POM to the total mass of particles [27].

### 3.8. Separation and Redispersion Process of **Fe_3_O_4_@1**

Figure S2 visually demonstrated the separation and redispersion process of the **Fe_3_O_4_@1** in water. Under the influence of an applied magnetic field, **Fe_3_O_4_@1** changed from a brown uniform dispersion to a transparent solution in water, and the nanocomposites were collected by a piece of a magnet, leaving no free **Fe_3_O_4_@1** visible. Namely, we carried out magnetic separation and found that all **Fe_3_O_4_@1** were collected by the magnet. The collected nanoparticles can be readily and reversibly dispersed by stirring after removing the magnetic field, and the process can be repeated.

### 3.9. Dye Adsorption Experiment

In this study, a typical cationic dye, MB, was selected to investigate the effect of dye concentration on adsorption. The concentration of the dye solution was determined by measuring the absorbance using a UV-Vis spectrophotometer.

**Figure 8:**
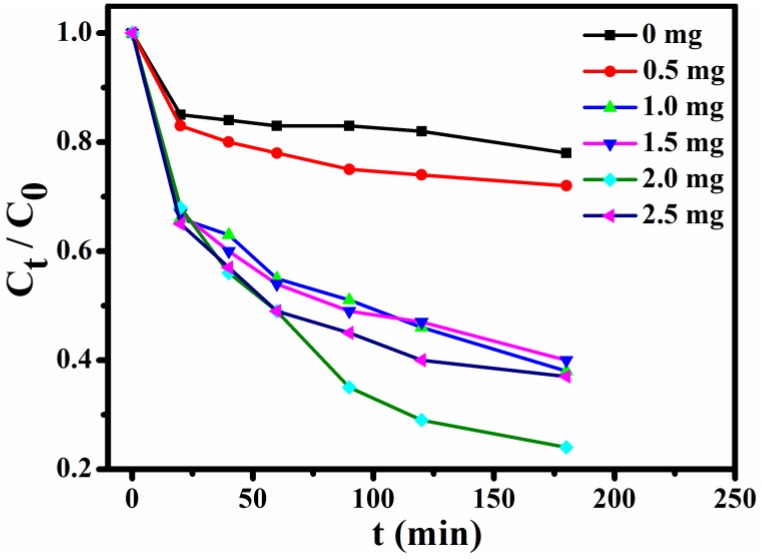
Effects of adsorbents usage on adsorption of MB.

Effects of adsorbents on adsorption:

In order to select the optimum usage amount of the adsorbent, a series of experiments with constant concentration of MB (10 mL, 15 mg L^−1^) solution were performed. Figure 8 shows the trends of normalized MB concentrations within 0–3 h at the given time intervals (C_t_, the MB concentration after adsorption at given time intervals; C_0_, the MB concentration before adsorption). These results of the incremental usage amount of **Fe_3_O_4_@1** (0, 0.5, 1.0, 1.5, 2.0 and 2.5 mg) indicate that the optimum usage amount is 2 mg.

**Figure 9:**
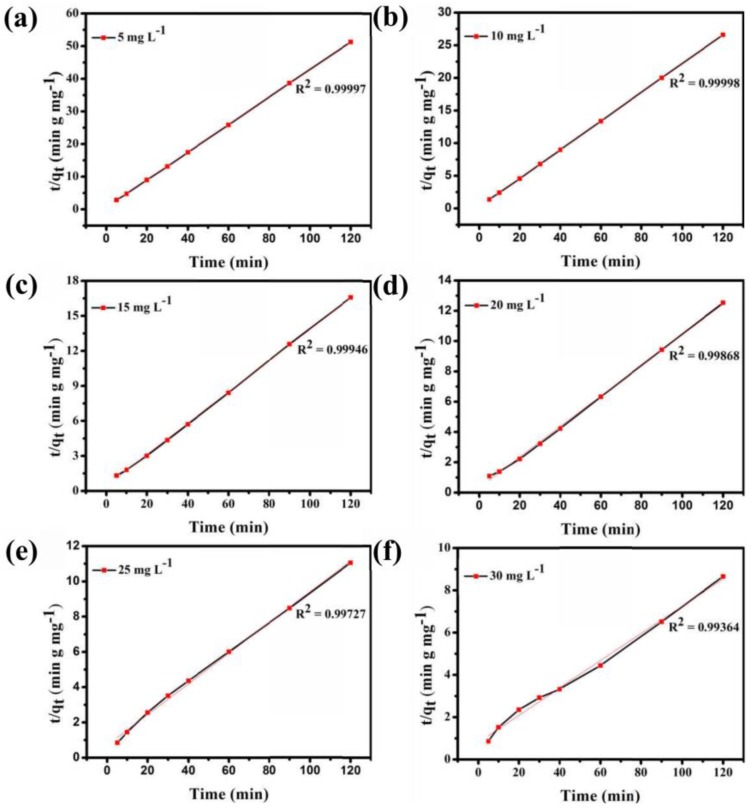
(**a**–**f**) Pseudo-second-order adsorption of MB at various initial concentrations in solution.

The effect of dye concentration: 

Figure S3 shows that the adsorption ability of **Fe_3_O_4_@1** is affected by the concentration of the dye solution. In the adsorption experiment, MB solutions of 5, 10, 15, 20, 25, and 30 mg L^−1^ were obtained by diluting 30 mg L^−1^ MB solution, and 2 mg **Fe_3_O_4_@1** was used as the adsorbent to remove MB. As seen in Figure S3, the adsorption efficiency of 10 mL of 5, 10, 15, 20, 25, and 30 mg L^−1^ MB solutions quickly reached 91.9%, 93.2%, 93.3%, 92.1%, 70.2%, and 83%, respectively, in the first 30 min. The adsorption efficiency of the MB solution reached 93.7%, 95.2%, 96.9%, 97.2%, 89.4%, and 95.5%, respectively, within 120 min. As exhibited in Figure 9, the UV-Vis spectroscopy results show that **Fe_3_O_4_@1** displays a perfect ability to remove MB. And the optimum concentration of the dye is 15 mg L^−1^. And a pseudo-first-order model and pseudo-second-order kinetic model were used to fit the experimental data (Figure 9). As expected, high correlations between the experimental data for the adsorption of MB by the nanocomposite and the pseudo-second-order kinetic model were indicated by the R^2^ values. These results strongly demonstrate that the great adsorption performance of **Fe_3_O_4_@1**. This is due to electron transfer and other chemical adsorption rather than simple adsorption of single molecules with the increase of MB concentration [28]. 

**Figure 10:**
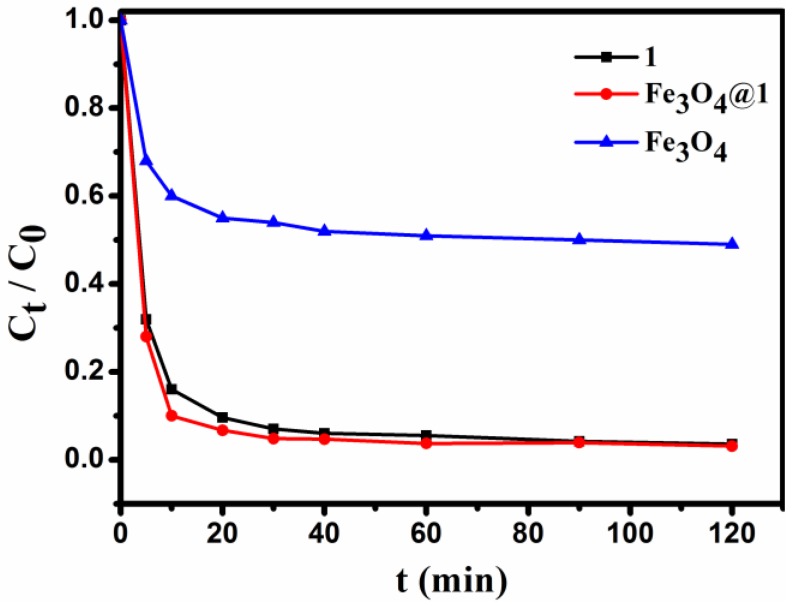
The changes in concentration of the MB dye solution.

Active site exploration:

For comparison with **Fe_3_O_4_@1**, the staring compounds **1** and Fe_3_O_4_ under the same condition was also tested. Typically, 2 mg of adsorbent is added to 10 mL of 15 mg L^−1^ dye solution under stirring conditions, and the concentration of the solution is detected for a period of time. As seen in Figure 10, **1**, Fe_3_O_4_ and **Fe_3_O_4_@1** were able to adsorb the MB in the dark, and the removal efficiency for **1**, Fe_3_O_4_ and **Fe_3_O_4_@1** were up to 96.5%, 51.4%, and 96.9%, respectively. Obviously, the Fe_3_O_4_ sample showed a lower adsorption capacity for MB. We speculated that the adsorption reaction of **Fe_3_O_4_@1** might be concentrated on **1**.

The reusability and stability of the composite material:

The reusability and stability of the materials have attracted considerable attentions. This will contribute to the process intensification, and minimize environmental burden [29,30]. To verify the stability of the **Fe_3_O_4_@1** and recover them in adsorption experiments, cycle tests of **Fe_3_O_4_@1** on removing MB were conducted. After each cycle, the adsorbent was simply centrifuged because it is insoluble in water. After the adsorbed MB **Fe_3_O_4_@1** was immersed absolute alcohol to release MB at room temperature, the regenerated **Fe_3_O_4_@1** was filtered and further washed with absolute alcohol. After that, regenerated **Fe_3_O_4_@1** was reused to investigate the adsorption capacity. Under stirring conditions, 10 mg of adsorbent was added to 20 mL of 15 mg L^−1^ MB solution.

**Figure 11:**
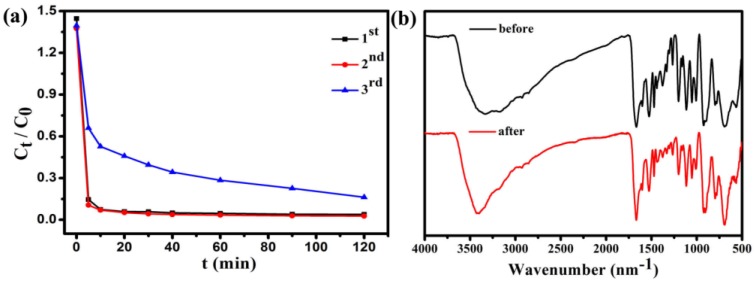
(**a**) Reuse performance of the **Fe_3_O_4_@1** adsorption experiments; (**b**) IR spectra of the fresh and recovered **Fe_3_O_4_@1**.

Figure 11 shows that the adsorption capacity of **Fe_3_O_4_@1** on MB. After two cycles, the regenerated adsorbent can still remove 96% of MB from the solution. The IR spectra of **Fe_3_O_4_@1** bulky samples collected from the adsorption experiments after two runs agreed well with the fresh samples, which indicated that the adsorbents remained intact (Figure 11b). These results show that the **Fe_3_O_4_@1** is reusable in adsorption experiments, which has potential application prospects in wastewater treatment.

**Figure 12:**
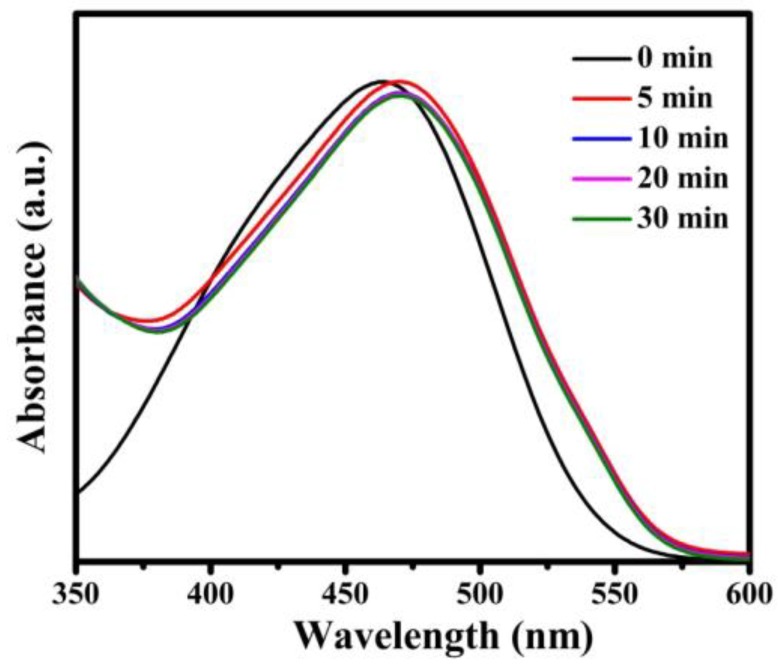
Adsorption spectra of the MO solution under the dark in presence of **Fe_3_O_4_@1**.

In order to further demonstrate the adsorption effect of **Fe_3_O_4_@1** on organic dyes, we explored a series of experiments in removing the different types of organic dyes, such as cationic dyes RhB, T, GV, FB and anionic dye MO. As shown in Figure S4, the absorption peak of each cationic dye decreased while time increased and the adsorption efficiency of RhB, T, GV and FB were 96.3%, 89.1%, 96.1%, and 94.5% in 180 min, 60 min, 20 min, and 180 min, respectively. Figure 12 indicates that the absorption peaks of anionic dye MO. The reason of the same adsorbent with different effects on removal of dyes is attributed to the electrostatic interactions between **Fe_3_O_4_@1** and cationic dye molecules, which have been verified by the pseudo-second-order kinetic model [28]. Accordingly, **Fe_3_O_4_@1** composite material is an adsorbent for cationic dyes in the dye-wastewater.

## 4. Conclusions

The development of removing organic dyes from wastewater has attracted increasing concerns. **Fe_3_O_4_@1** has been synthesized by combining Fe_3_O_4_ and polyoxometalate. The morphology and structural analyses reveal the narrow particle size distribution, with an average diameter 19.1 nm. The magnetic characterization shows that **Fe_3_O_4_@1** has superparamagnetic or soft ferromagnetic behavior. The **Fe_3_O_4_@1** has selective adsorption behavior toward cationic organic dyes: MB, RhB, T, GV, and FB, with adsorption efficiencies of 96.9%, 96.3%, 89.1%, 96.1%, and 94.5%, respectively. Importantly, the nanocomposite particle **Fe_3_O_4_@1** exhibits recyclability and stability. After two cycles, the regenerated adsorbent remained intact, and could still remove 96% of MB from the solution. The basic research and application of **Fe_3_O_4_@1** in the magnetic adsorption field are promising. Future work will concentrate on improving the activity and selectivity for organic dyes and the synthesis of novel nanocomposites.

## Figures and Tables

**Table 1:** Summary of crystal data and refinement results for compound **1**.

Crystal Data	1
Empirical formula *Formula weight*	C_32_H_58_Cu_2_Mo_5_N_16_O_37_P_2_ 1909.52
crystal system	Triclinic
space group	*P-1*
Temperature (K)	296(2)
*a* (Å)	14.0430(7)
*b* (Å) *c* (Å) *α* (deg)	14.6752(7) 18.3928(9) 71.4980(10)
*β* (deg) *γ* (deg) Volume (Å^3^) Z	69.5040(10) 63.3060(10) 3113.5(3) 2
Calculated density (g cm^−3^)	2.037
Absorption coefficient (mm^−1^) Crystal size (mm^3^)	1.804 0.41 × 0.33 × 0.25
Theta range for data collection (deg)	1.58–25.00
*F*(000)	1876
Limiting indices	−16 ≤ *h* ≤ 16, −14 ≤ *k* ≤ 17, −14 ≤ *l* ≤ 21
*R_int_*	0.0165
parameters	833
Reflections collected/unique	10869/9512
Final R indices [*I* ≥ 2σ(*I*)]	*R*_1_ = 0.0318, *wR*_2_ = 0.0902
R indices (all data)	*R*_1_ = 0.0379, *wR*_2_ = 0.0931
Largest diff. peak and hole (e Å^−3^)	2.103, −0.677

## Supplementary Materials

The following are available online at http://www.mdpi.com/2079-4991/8/9/710/s1, Figure S1: TEM analyses, (a) morphology image of Fe_3_O_4_; (b) morphology image of **Fe_3_O_4_@1**; Figure S2: Photographs of the dispersion-collection process of **Fe_3_O_4_@1** in water; Figure S3: Adsorption capacity of **Fe_3_O_4_@1** with various initial concentrations of MB in solution; Figure S4: Adsorption spectra of the RhB solution (a); the T solution (b); the GV solution (c) and the FB solution (d) under the dark in presence of **Fe_3_O_4_@1**.
