# Supplementary material for: A Magnetic Adsorbent for the Removal of Cationic Dyes from Wastewater
Source: Nanomaterials (Basel). 2018 Sep 10;8(9):710. doi: 10.3390/nano8090710 (PMC6163381; doi:10.3390/nano8090710)
Supplement: Supplementary file 1 [file nanomaterials-08-00710-s001.pdf]

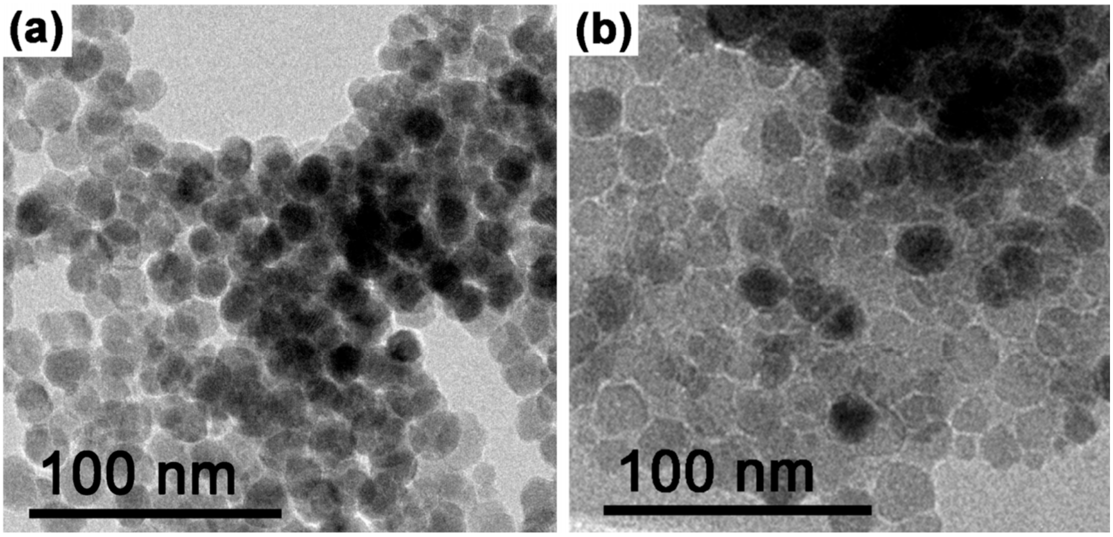

Figure S1. TEM analyses, (**a**) morphology image of Fe_3O_4 and (**b**) morphology image of **Fe_3O_4@1**.

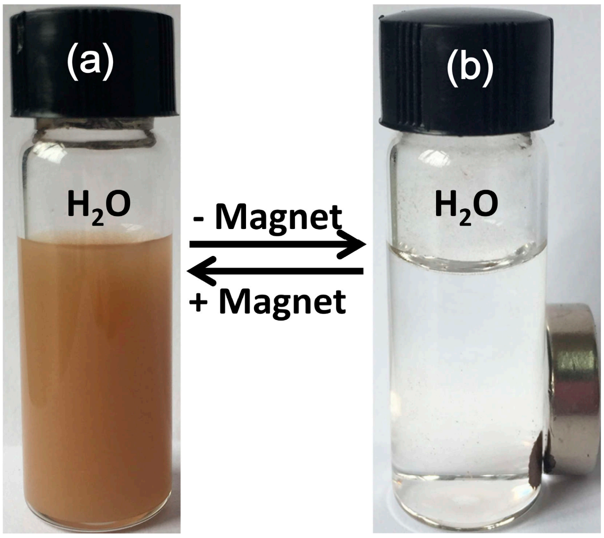

Figure S2. Photographs of the dispersion-collection process of **Fe_3O_4@1** in water.

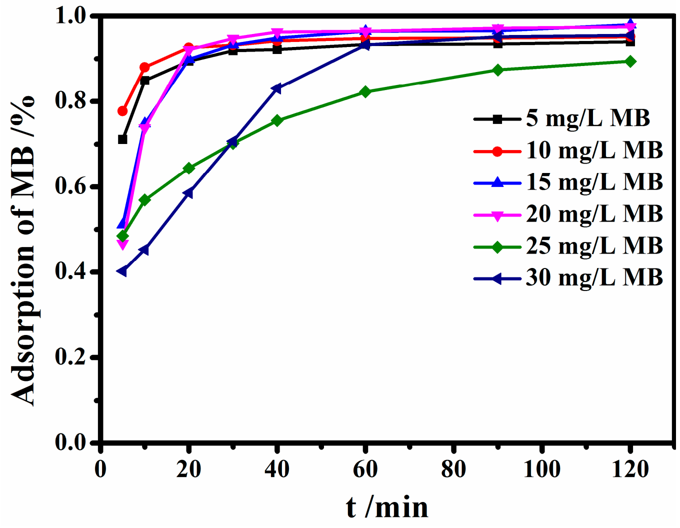

Figure S3. Adsorption capacity of **Fe_3O_4@1** with various initial concentrations of MB in solution.

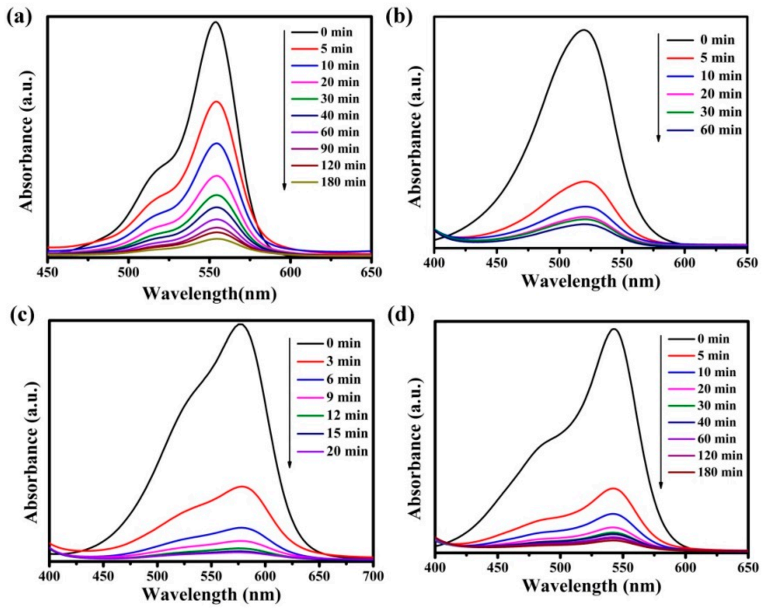

Figure S4. Adsorption spectra of the RhB solution (a); the T solution (b); the GV solution (c) and the FB solution (d) under the dark in presence of **Fe_3O_4@1**.
